# Retrospect on the Use of Raw Meat in the Diarrhœa of Weaned Children

**Published:** 1859-08

**Authors:** J. F. Weisse

**Affiliations:** Director of the Children’s Hospital at St. Petersburgh


### RETROSPECT ON THE USE OF RAW MEAT IN THE DIARRHŒA OF WEANED CHILDREN.

BY DR. J. F. WEISSE, DIRECTOR OF THE CHILDREN'S HOSPITAL AT ST. PETERSBURGH.

Seventeen years have now elapsed since I first directed the attention of the profession to this invaluable remedy in the above disease;* but it was not until I had five years later treated of the subject at greater length,† that it came into more general use. Soon after the publication of the latter paper, I received from the esteemed editor of the Journal just now quoted, Dr. Behrend, of Berlin, a letter containing the following passage: "You have no idea what interest your communication on diarrhœa ablactatorum and on the use of raw meat has excited; we now use the remedy extensively."

Not long after, Dr. Behrend inserted in the sixth volume of his Journal a letter of M. Marotte, Physician of the Central Bureau of the Parisian Hospitals, from which it appeared that this subject had attracted great attention also in the French metropolis. The author of this letter, which is addressed to Dr. Trousseau, has moreover had the kindness to suggest a theory explanatory of the results I have obtained. From this time the meat cure was generally received, and its utility admitted on all sides. Of the numerous favorable reports recently published, I cannot forbear literally transcribing that contributed by Dr. Eichelberg, because the author has given to the subject the appreciation it deserves. He says: "In consequence of the shortness of the time which has elapsed since this article of diet was first recommended, I have, it is true, only a limited number of observations (somewhat more than twenty) before me, but they all corroborate the remarkable advantages of the plan proposed. It is only in exceptional instances that such children refuse raw meat—the great majority, in fact, consume it with manifest relish. I have observed two very striking cases where

---

* Oppenheim's Journal f. d. gesammte Medizin, Bd. xiii. page 393. Hamburgh, 1840.

† Journal f. Kinderkrankheiten, Bd. iv. pp. 99–104. Berlin, 1845.

the children for several weeks readily partook of this food with the most beneficial results, and at the end of that time suddenly refused it. Natural instinct seems in such examples to be unmistakable, as in the case of sick dogs, which eat grass. The want of osmazome made the children greedily consume the raw meat, but, with the cessation of the want, the desire for that principle disappeared."*

As Herr Eichelberg, moreover, has expressly indicated the diarrhœa which sets in soon after the weaning of children (according to my observations usually in two or three weeks after that event), as the affection in which the raw meat cure is attended with certain success, so I have also, in recommending this mode of treatment, confined myself to the same disease; and now, after nearly twenty years' experience, maintain, that raw, scraped beef, to the exclusion of all other medication, is a true specific in this destructive diarrhœa. I therefore consider a remark made by Charles Hogg, in recommending the well-known "beef-tea" of the English, to be quite erroneous. Thus he says: "Beef-tea is an excellent, nourishing, and easily digestible article of food, and completely replaces the juice of meat recommended by Weisse, of St. Petersburgh, obtained by scraping raw flesh."† I have in raw beef discovered, not an article of food for children, but a remedy against the diarrhœa in question; nor have I spoken of the juice to be obtained by scraping meat, but the muscular substance itself must be given to the children, having, however, previously been sufficiently comminuted, either by scraping with a knife, or by means of a grater, in order that it may be swallowed without trouble. But the principal point is, that the muscular substance itself, and not merely its juice, should be conveyed into the digestive tube. The English beef-tea has as little beneficial effect on diarrhœa ablactatorum as Liebig's excellent decoction of meat. Both these fluid aliments appear, precisely because they are fluid, to pass too quickly through the intestinal canal; while the meat in substance remains longer in the tube, and by its mechanical irritation may stimulate digestion, and it may, perhaps, also neutralize the acidity of the gastric juice. Nor can I participate in Dr. Beer's sanguine hope that raw grated beef may be destined one day to dislodge cod-liver oil from the Materia Medica.‡ Each of these excellent remedies has its definite sphere of medical action in the diseases of children; raw beef in the diarrhœa

* Journal f. Kinderkrankheiten, Bd. iv. pp. 99–104. Berlin, 1845.

† Journal f. Kinderkrankheiten, Bd. xiv. p. 113, in the notice of Hogg's treatise "On the Management of Infancy," etc.

‡ Journal f. Kinderkrankheiten, Bd. xiv. p. 238.

ablactatorum, cod-liver oil in rachitic affections, with and without atrophy.

In St. Petersburgh, the meat cure in the affection of children under consideration has become, so to speak, completely naturalized; and this has taken place rather through oral communication, and in consequence of the favorable results of the treatment, than from any paper or essay, as I have never published anything in that capital upon the subject. Most of my colleagues have now for several years made use of it, and they all assure me that they have obtained very satisfactory results, even in cases where the employment of other established remedies appeared to hold out no hope of cure. I have myself seen this treatment adopted in about two hundred children, and, in the majority, with the desired effect, provided recourse was had to it at the proper time. I say at the proper time, for if the disease has already advanced too far, and, particularly, if it has assumed the form of the so-called gastro-malaria, it is only in exceptional instances that we shall obtain a cure. But even in this case there is no other remedy so calculated to allay the most tormenting symptoms, the tantalizing thirst, and the vomiting, as the raw beef. This beneficial effect is produced even after a raw meal.

But it has recently been stated, as I have already publicly remarked,* that in many children saved by the meat cure, tape-worm, and it is worthy of note, always the tænia solium, that is precisely the species which is not indigenous in St. Petersburgh, has shown itself. A Dr. Braun† has felt himself called upon to question this statement; two years later, however, an undoubted authority on this subject appeared in favor of the facts reported by me. Professor D. Von Siebold of Munich, says, in the last page of his interesting work, "Ueber die Band und Blasenwuemer," Leipsic, 1854: "We can no longer be surprised, or consider their statements fabulous, when physicians report that tape-worms have been found in certain patients after the use of raw meat prescribed as a remedy;" and in the note upon this passage, he adds: "Compare on this subject Weisse's communications, which, notwithstanding Braun's objections, are worthy of all credit." Herr Von Siebold directs particular attention to the fact that in every instance it was the tænia solium which was passed; and he considers it probable that this species of tape-worm, which is not indigenous in St. Petersburgh, may have been conveyed thither

* Journal f. Kinderkrankheiten, Bd. xvi. p. 384. 1851.

† Ibid., Bd. xviii. p. 78, 1852; and in Froriep's Togesberichten. 1852.

in the undeveloped state in the flesh of oxen, brought from Tscherkask and Podolia.

Only a few weeks before my departure from St. Petersburgh, in June of the present year, a tape-worm, more than four feet long, was sent to me by a colleague, to whom I had warmly recommended the meat cure in the case of a child, aged eighteen months, who had suffered from the diarrhœa in question, and was already very much run down, which worm was passed after the use of the ethereal oil of male fern. This remedy was administered in consequence of the child, who had long ceased to get the raw meat, and was cured of the diarrhœa, having repeatedly passed joints of tape-worm. The attendant physician had already correctly diagnosed the worm to be the tænia solium; I found that it was voided with the head, on which the suckers were plainly distinguishable under the microscope.

I should not omit to state, that in the Children's Hospital under my care, in the diarrhœas of older children, into which the element of dentition no longer enters, and which so largely contribute to fill the lists of mortality, raw meat has been repeatedly and unsuccessfully tried. These cases of diarrhœa generally depend upon ulcerations in the intestinal canal.

Lastly, I may be allowed to call the attention of the meeting to as palatable a remedy as raw beef, in the lientery of adults; I allude to oysters. In two cases, an amount of experience, which, I must admit, goes for nothing, I saw the patients cured by the moderate use of these mollusca. From eight to twelve oysters were taken daily in two meals.—*Dublin Quarterly Journal*, Feb., 1858, from *Journal fur Kinderkrankheiten*, Jan. and Feb., 1858.

